# Fish oil administration mediates apoptosis of Walker 256 tumor cells by modulation of p53, Bcl-2, caspase-7 and caspase-3 protein expression

**DOI:** 10.1186/s12944-015-0098-y

**Published:** 2015-08-25

**Authors:** Gina Borghetti, Adriana Aya Yamaguchi, Julia Aikawa, Ricardo Key Yamazaki, Gleisson Alisson Pereira de Brito, Luiz Claudio Fernandes

**Affiliations:** Centro de Estudos da Biodiversidade, Universidade Federal de Roraima, Campus Paricarana Avenida Capitão Ene Garcez, 2413, Bairro Aeroporto Cep: 69310-000, Boa Vista, Roraima Brazil; Instituto de Pesquisa Pelé Pequeno Príncipe, Hospital Pequeno Príncipe, Curitiba, Paraná Brasil; Departamento de Fisiologia, Laboratório de Metabolismo Celular, Universidade Federal do Paraná, Curitiba, Paraná Brasil; Universidade Federal da Fronteira Sul, Laranjeiras do Sul, Paraná Brasil; Universidade Federal da Integração Latina Americana, Foz do Iguaçu, Parana Brazil

**Keywords:** Fish oil, Caspases, Apoptosis, Walker 256 tumor, n-3 PUFA, Protein expression

## Abstract

**Background:**

Several studies have been shown pro-apoptotic effects of fish oil (FO)*,* rich in n-3 polyunsaturated fatty acids (n-3 PUFA) on cancer cells. Nevertheless, few in vivo experiments have provided data of its ability on apoptosis protein expression in tumor tissue. Thus, in this study we investigate the effect of FO supplementation on apoptosis protein expression in Walker 256 tumor bearing rats. Male Wistar rats were randomly assigned to three groups: fed with regular chow (W); fed regular chow supplemented with FO (WFO) or coconut fat (WCO) (1 g/kg body weight/daily). After thirty days, all animals were inoculated subcutaneously with Walker 256 tumor cells.

**Findings:**

Protein expression was done by western blotting in Walker 256 tumor tissue samples. FO decreased the Bcl-2/Bax ratio (*p* < 0.05) and increased the p53 (*p* < 0.05), cleaved caspase-7 (*p* < 0.05) and cleaved caspase-3 (*p* < 0.05) in Walker 256 tumor tissue*.*

**Conclusions:**

Our data suggest that the pro-apoptotic effect of FO in Walker 256 tumor is related with specifics cleaved caspases.

## Background

N-3 polyunsaturated fatty acids family presents several biological properties, including anti-cancer development and cachexia [1]. We previously reported that administration of fish oil (FO), rich in n-3 polyunsaturared fatty acids (n-3 PUFA), caused reduction of cell proliferation and increased apoptosis in Walker 256 tumor cells [2].

Apoptosis is an important process of programmed cell death with precise biochemical and genetic pathways. In cancer cells, apoptosis is reduced by different mechanisms, which leads to tumor development [3]. The apoptosis signaling occurs by several independent routes that converge to activation of caspases, a family of cysteine proteases that act as regulators of initiation and execution of apoptosis. This activation can be controlled by the Bcl-2 family proteins, which have pro-apoptotic and antiapoptotic effects [4, 5]. Furthermore, the p53 protein can interact with the proteins of Bcl-2 family and induce apoptosis [6, 7]. The mechanisms underlying n-3 PUFA pro-apoptotic effect in cancer cells is still unknown.

There are few in vivo experiments providing data on the ability of FO to alter the apoptosis protein expression in tumor tissue. In this report, we bring new information in this subject once we examinate Bcl-2/Bax ratio, p53, caspase-7 and caspase-3 protein expression in Walker 256 tumor tissue after FO administration.

## Materials and methods

### Animals and study design

All experimental procedures were approved by The Local Ethics Animal Experiment Committee, and were carried out in accordance with the ethical principles established by the Experimental Brazilian Council (COBEA). Male Wistar rats 70 days old were maintained under controlled temperature (22 ± 3 °C), 12 h/12 h light/dark cycle and received regular chow diet (Nuvilab CR-1, Nuvital) ad libitum. The content of commercial regular chow indicates: carbohydrate 63,4 % kcal, protein 25,6 % kcal and lipid 11 % kcal. The animals were randomized into three groups: Walker 256 tumor-bearing rats fed with regular chow (W); Walker 256 tumor-bearing rats fed with regular chow plus fish oil supplementation (WFO) or coconut fat (WCO). The fish oil was kindly donated by *Herbarium Foundation* and the coconut fat was purchased from *Refino de oleos*. The fatty acid composition of the chow, fish oil (FO) and coconut (CO) was determined by high-performance liquid chromatography (% total fatty acid) (Table 1). The WFO and WCO groups received 1 g/kg/day of its respective oils, administered as single bolus using a micro pipette until the end of experiment (45 days of supplementation). Body mass from all animals was monitored every 2 days during the experiment.

**Table 1:** Fatty acid profile of regular chow, FO, CO and tumor tissue

Fatty acids (g/100 g total fatty acids)	Regular chow	Fish oil	Coconut fat	Tumor tissue		
				W	WFO	WCO
Lauric acid (12:0)	1.3 ± 0.3	4.8 ± 0.1	45.3 ± 3.1	4.6 ± 1.4	2.3 ± 0.4	5.4 ± 1.8
Miristic acid (14:0)	-	9.9 ± 0.1	17.0 ± 1.9	1.8 ± 1.8	2.5 ± 1.4	2.2 ± 1.3
Palmitic acid (16:0)	13.7 ± 0.9	16.7 ± 0.2	25.0 ± 2.8	19.2 ± 0.2	16.9 ± 0.2	15.3 ± 0.3
Stearic acid (18:0)	2.4 ± 0.3	1.9 ± 0.3	1.9 ± 0.2	12.6 ± 0.4	5.7 ± 1.2	8.5 ± 1.1
Oleic acid (18:1n-9)	20.0 ± 0.1	10.6 ± 0.1	7.9 ± 0.2	18.9 ± 0.7	22.6 ± 0.2	22.6 ± 0.5
Linoleic acid (18:2n-6)	56.0 ± 0.8	11.6 ± 0.1	2.0 ± 1.2	20.5 ± 0.5	35.4 ± 0.1	33.7 ± 1.1
α -Linolenic acid (18:3n-3)	6.0 ± 0.6	-	0.9 ± 0.1	0.5 ± 0.1	2.0 ± 0.2	1.6 ± 1.5
Arachidonic acid (20:4n-6)	0.3 ± 0.2	0.7 ± 0.1	-	19.0 ± 0.4	6.5 ± 0.3	8.5 ± 0.6
Eicosapentaenoic acid (20:5n-3)	0.2 ± 0.1	23.9 ± 0.6	-	0.3 ± 0.3	2.0 ± 0.7*	0.4 ± 0.3
Docosahexaenoic acid (22:6n-3)	-	19.8 ± 0.8	-	2.5 ± 1.1	4.1 ± 1.8*	1.8 ± 1.7
n-6 / n-3 PUFA ratio	9.0	1.4	8.4	12.0	5.2	11.0

Fatty acid composition of regular chow, fish oil (FO), coconut fat (CO) and tumor tissue by high performance liquid chromatographer. Data are mean ± SEM (*n* = 9) of Walker 256 tumor-bearing rats (W), Walker 256 tumor-bearing rats supplemented with fish oil (WFO) and coconut fat (WCO) * *p* < 0.05 vs. W and WCO group

### Walker 256 tumor cell

Walker 256 tumor is a carcinosarcoma that has been maintained in our Laboratory. The tumor cells were obtained from a rat ascitic fluid by intraperitoneal passages as described elsewhere [8]. The percentage of viable cells was established by trypan blue solution (1 %) using a Neubauer chamber. When animals reached 100 days of age, all groups (W; WFO; WCO) were inoculated subcutaneouly in the right flank with 1 mL of a sterile suspension of 1x10^8^ Walker tumor cells obtained from an ascitic tumor-bearing rat.

### High-performance liquid chromatography

The quantification of fatty acid composition in the chow, fish oil, coconut fat and tumor tissue was performed by high-performance liquid chromatography (HPLC) as described elsewhere [1]. The n-6 PUFA: linoleic acid (LA); arachidonic acid (AA) and n-3 PUFA: α-linolenic acid (ALA), eicosapentaenoic acid (EPA), docosahexaenoic acid (DHA) were used to determined n-6/n-3 PUFA ratio.

### Western blotting

Tumor tissue samples (100 mg) were homogenized in lysis buffer plus protease inhibitor tablet (Sigma-Aldrich). Protein concentration was determined using Bradford assay [9]. Sample proteins (45 μg) were loaded onto a range from 8–15 % Polyacrylamide Gel Electrophoresis and transferred onto nitrocellulose membrane by electro blotting under wet conditions (Mini Trans blot Bio-Rad). The membranes were incubated overnight at 4^o^ C individually with the following antibodies: anti-p53, anti-Bcl-2, anti-Bax, anti-β-actin (Santa Cruz Biotechnology), anti-caspase-7, anti-cleaved caspase-7, anti-caspase-3, anti-cleaved caspase-3, anti-PARP-1, anti-cleaved PARP-1 (Cell Signaling Technology) at 1:1000 dilution. Then, they were incubated with their secondary antibody conjugated horseradish peroxidase (Santa Cruz Biotechnology) for two hours at room temperature at 1:6000 dilution. Then they were exposed to Kodak® film with chemiluminescent substrate (Super Signal System Pierce) and the resulting bands were analyzed and quantified by Image J® (National Institute of Health). β-actin was used as housekeeping.

### Statistical analysis

The statistical analysis was performed by one-way analysis of variance (ANOVA), followed by post hoc Tukey test, using GraphPad Prism software (GraphPad Inc.). All data are reported as mean ± standard error of the mean *a*nd value of *p* < 0.05 was taken to indicate statistical significance.

## Results

**Fig. 1:**
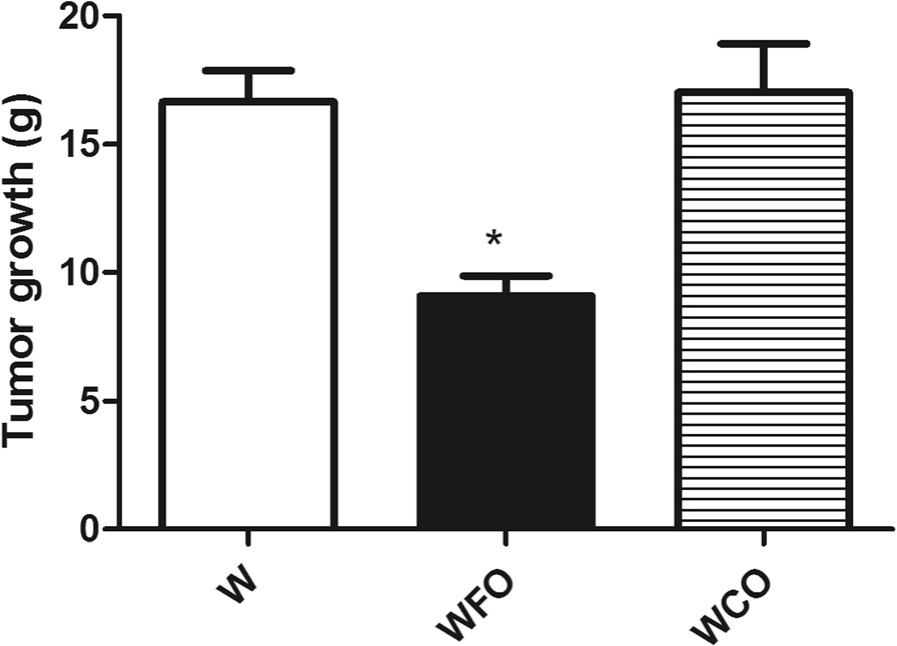
Tumor growth (g) of Walker 256 tumor-bearing rats (W), Walker 256 tumor-bearing rats supplemented with fish oil (WFO) and coconut fat (WCO). Data are mean ± SEM (*n* = 15) * *p* < 0.05 vs. W and WCO group

**Fig. 2:**
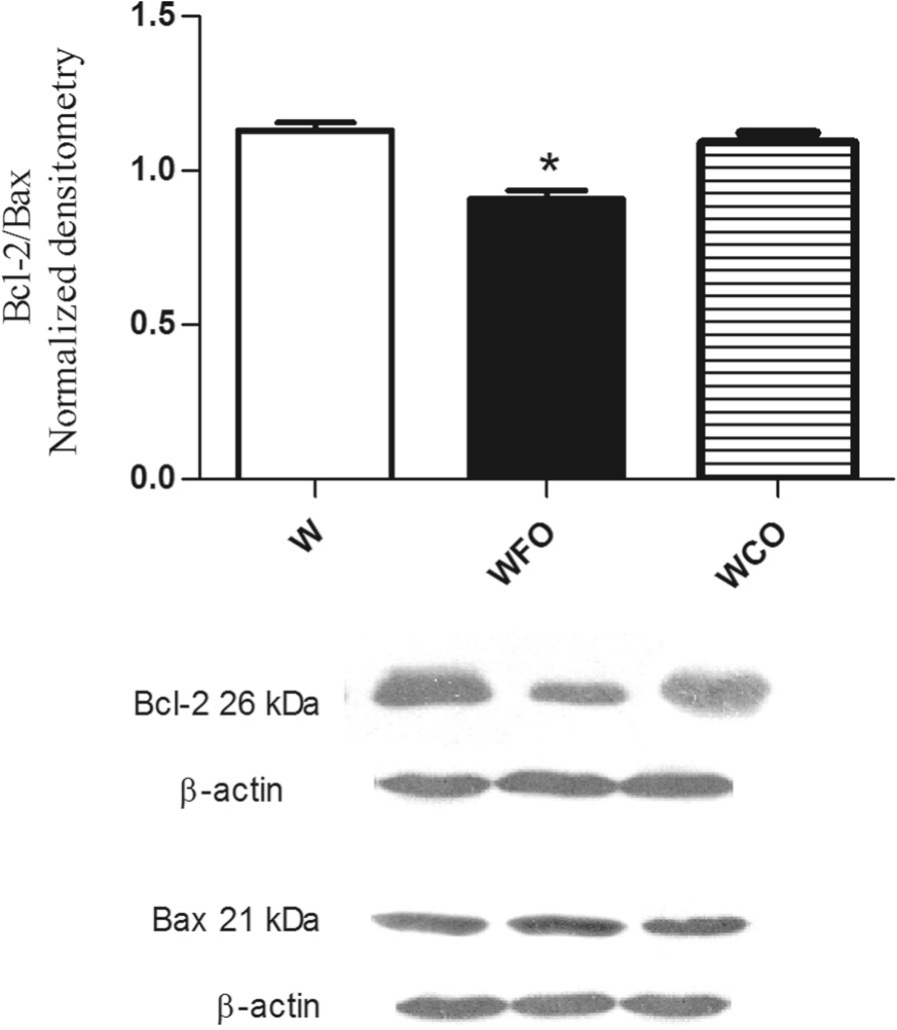
Protein expression of Bcl-2/Bax ratio in the Walker 256 tumor tissue. Anti-β-actin was used as a loading control to normalize the data. Data are mean ± SEM (*n* = 7) of Walker 256 tumor-bearing rats (W), Walker 256 tumor-bearing rats supplemented with fish oil (WFO) and coconut fat (WCO) * *p* < 0.05 vs. W and WCO group

**Fig. 3:**
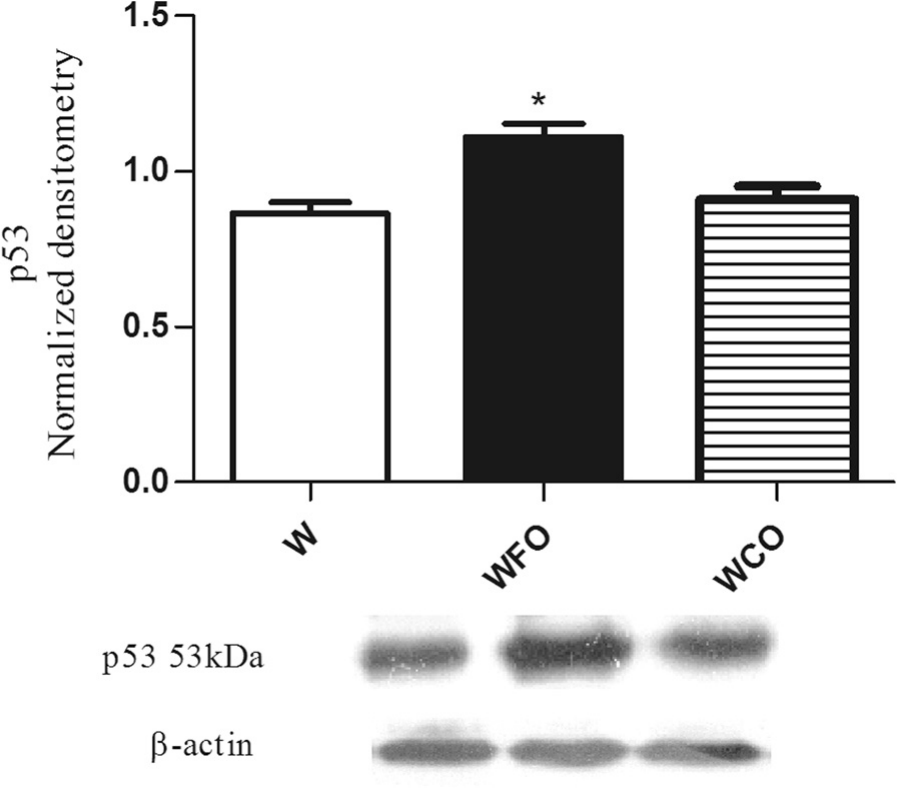
Protein expression of p53 in the Walker 256 tumor tissue. Anti-β-actin was used as a loading control to normalize the data. Data are mean ± SEM (*n* = 7) of Walker 256 tumor-bearing rats (W), Walker 256 tumor-bearing rats supplemented with fish oil (WFO) and coconut fat (WCO) * p < 0.05 vs. W and WCO group

**Fig. 4:**
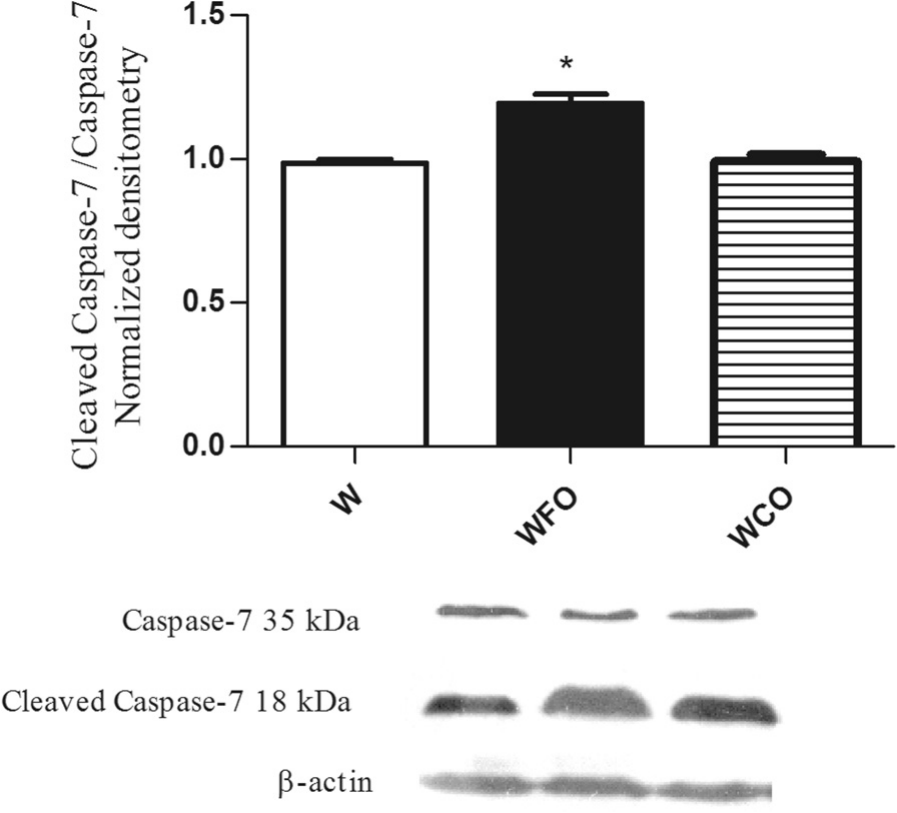
Protein expression of cleaved caspase-7 in the Walker 256 tumor tissue. Anti-β-actin was used as a loading control to normalize the data. Data are mean ± SEM (*n* = 7) of Walker 256 tumor-bearing rats (W), Walker 256 tumor-bearing rats supplemented with fish oil (WFO) and coconut fat (WCO) * *p* < 0.05 vs. W and WCO group

**Fig. 5:**
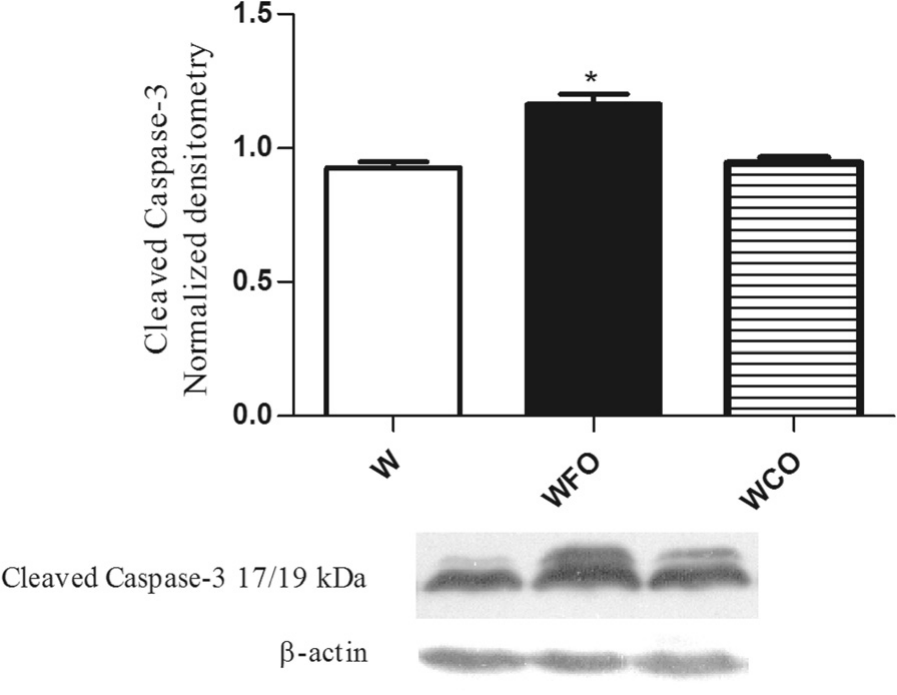
Protein expression of cleaved caspase-3 in the Walker 256 tumor tissue. Anti-β-actin was used as a loading control to normalize the data. Data are mean ± SEM (*n* = 7) of Walker 256 tumor-bearing rats (W), Walker 256 tumor-bearing rats supplemented with fish oil (WFO) and coconut fat (WCO) * *p* < 0.05 vs. W and WCO group

**Fig. 6:**
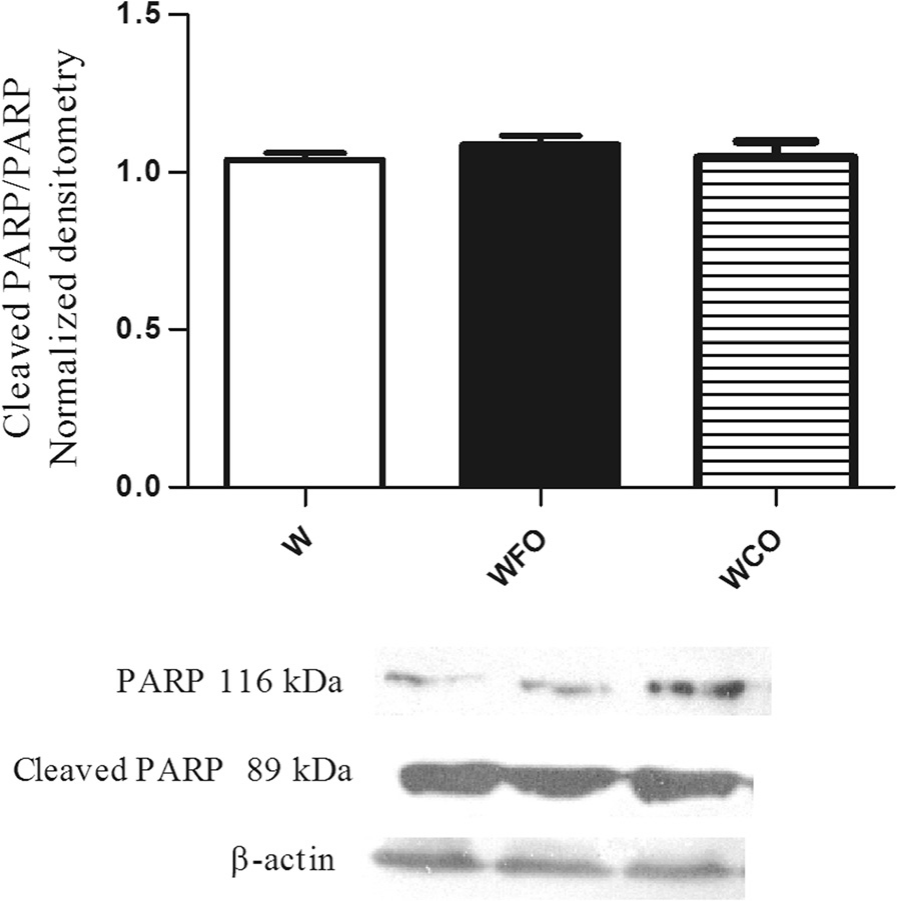
Protein expression of PAPR-1 in the Walker 256 tumor tissue. Anti-β-actin was used as a loading control to normalize the data. Data are mean ± SEM (*n* = 7) of Walker 256 tumor-bearing rats (W), Walker 256 tumor-bearing rats supplemented with fish oil (WFO) and coconut fat (WCO) * *p* < 0.05 vs. W and WCO group

FO supplementation decreased the n-6/n-3 PUFA ratio in tumor tissue by 2 fold (Table 1) and reduced the tumor weight by 47 % (W 16.9 ± 1.2 vs. WFO 9.0 ± 0.8 vs. WCO 17.1 ± 2.1) (Fig. 1) when compared with Walker 256 tumor-bearing rats fed with regular chow group (W) or WCO group (*p* < 0.05). Tumors from Walker 256 tumor-bearing rats fed with regular chow plus fish oil supplementation group (WFO) had a significant decrease of 20.3 % (W 1.13 ± 0.03 vs. WFO 0.90 ± 0.03 vs. WCO 1.09 ± 0.03) from Bcl-2/Bax ratio when compared with W or WCO group (*p* < 0.05) (Fig. 2). FO supplementation also increased the protein expression of p53 by 29 % (W 0.86 ± 0.04 vs. WFO 1.11 ± 0.04 vs. WCO 0.91. ± 0.04) (*p* < 0.05) (Fig. 3), cleaved caspase-7 by 21.4 % (W 0.98 ± 0.01 vs. WFO 1.19 ± 0.03 vs. WCO 0.99 ± 0.03) (*p* < 0.05) (Fig. 4) and cleaved caspase-3 by 26 % (W 0.92 ± 0.02 vs. WOP 1.16 ± 0.04 vs. WCO 0.94 ± 0.02) (*p* < 0.05) (Fig. 5) in tumor tissue when compared with W and WCO. FO supplementation did not modify the PARP-1 protein expression in tumor tissue (W 1.03 ± 0.02 vs. WFO 1.08 ± 0.03 vs. WCO 1.04 ± 0.05) (Fig. 6). There was no difference in the tumor protein expression of WCO when compared with W group (*p* > 0.05).

## Discussion

Previous studies have shown that diets rich in n-3 PUFAs decrease tumor growth in Walker 256 tumor-bearing rats [1, 2, 10]. The increase of apoptosis by n-3 PUFA is one of the possible mechanisms involved in this process [2]. Our data support these hypothesis, once FO supplementation altered the expression of key apoptotic proteins in the Walker 256 tumor tissue. Furthermore, literature suggests that n-6/n-3 PUFA ratio reduction leads to anti-tumor activity [11, 12]. Incorporation of n-3 PUFA in the tumor cell membrane may change apoptosis cell signaling, such as caspase-3 [13]. In addition, Willliams et al. also showed effects of n-6/n-3 PUFA ratio on prostate tumors [14]. Several studies have suggested that DHA may influence p53 expression, associated with the activation of caspase-9 and caspase-3 in Reh cells [6]. Besides, n-3 PUFAs may activate important molecules that signal to carcinogenesis, such as Bcl-2 family proteins [15–17]. We found that Bcl-2 expression, an antiapoptotic protein, is inversely related to p53 expression, indicating that the increase of p53 may decrease the Bcl-2 protein, which corroborate with previous studies [18]. P53 protein expression may also play an important role in n-3 PUFA’s anti-proliferative effects. Moreover we did not detect any change in Bax expression, which is a pro-apoptotic protein. Even without Bax expression alteration, the Bcl-2/Bax ratio was found lower in the WFO group, which suggests a favorable balance to increase apoptosis.

In order to seek further events in the molecular signaling of apoptosis we check the caspases activity in the Walker 256 tumor. Caspases-7 and caspase-3 are both executioner caspases and share common roles in apoptosis, where PARP-1 is the substrate cleaved by them [19, 20]. During cell death, detection of the cleaved PARP - 1 is regarded to be a hallmark of apoptosis [19, 21]. Members of PARP family can play a role in both pro - and anti - tumor process, depending on circumstances. Recent findings have suggested the involvement of PARP-1 on multiple tumorigenesis pathways, including cell proliferation and invasion, however, the exact mechanisms remain unclear [22, 23]. In this study, we found that FO is involved in the up regulation of apoptosis in Walker 256 tumor by increasing caspase-7 and caspase-3 activities. Besides PARP-1 is detected during apoptotic pathways in various cells’ type. Once our data did not show difference in PARP-1 expression, it is possible that PARP-1 activity is also involved in non- apoptotic death pathway in Walker 256 tumor cell. Although Caspase-3 and Caspase-7 have specifities in cleaving PARP-1, we propose that others caspases substrates may be involved during the execution of apoptosis. However, further research is needed to investigate the role of PARP-1 in Walker 256 tumor cell. Interestingly, we did not detect the expression of procaspase-3 in Walker 256 tumor by western blotting and studies are currently being conducted by our group to better understand this feature.

One major limitation of our experimental design is that we do not know which of the fish oil components is responsible for our findings in Walker 256 tumor. The beneficial effects of fish oil seem to be due to its high content of n-3 PUFA (combined: DHA and EPA) and the decrease of n-6/n-3 PUFA ratio. However, the molecular mechanisms by which DHA or EPA alone may affect the Walker 256 tumor is still unclear and need future studies.

## Conclusions

In summary, our findings suggest that FO induce apoptosis by p53, Bcl-2, caspase-7 and caspase-3, but not Bax and PARP-1 in Walker 256 tumor. This pro-apoptotic effect is related with caspase pathways and may be also associated with reduction of n-6/n-3 PUFA ratio.

## Abbreviations

FO: Fish oil
CO: Coconut fat
PUFA: Polyunsaturated fatty acid
SDS-PAGE: Sodium dodecyl sulfate-polyacrylamide gel electrophoresis
PARP-1: Poly(ADP-ribose) polymerase-1
